# The impact of residential segregation on life satisfaction among rural-urban migrants in China: the mediating role of social capital

**DOI:** 10.3389/ijph.2026.1609726

**Published:** 2026-08-06

**Authors:** Jingyao Hou, Zhongshan Yue

**Affiliations:** 1 School of Humanities and Social Science, Xi’an Jiaotong University, Xi’an, China; 2 School of Public Policy and Administration, Xi’an Jiaotong University, Xi’an, China

**Keywords:** China, life satisfaction, residential segregation, rural-urban migrants, social capital

## Abstract

**Objectives:**

Rapid urbanization in China has been accompanied by residential segregation, which may influence wellbeing of rural-urban migrants (RUMs). This study aims to investigate the impact of residential segregation on life satisfaction among RUMs, with particular attention to the mediating role of social capital.

**Methods:**

We screened 1,255 participants who met the inclusion criteria from the 2016 China Labor-force Dynamics Survey to investigate the relationship between residential segregation and life satisfaction using ordinary least squares regression and instrumental variable estimation. The mediating role of social capital was evaluated by mediation analysis.

**Results:**

Residential segregation has a significant negative impact on life satisfaction of RUMs. This effect is partially mediated by cognitive social capital, whereas no mediating effect is observed for structural social capital. Among the dimensions of cognitive social capital, neighborhood trust and reciprocity are more significant mediators than familiarity.

**Conclusion:**

Residential segregation undermines life satisfaction among RUMs partly through weakening cognitive social capital. Policies aimed at reducing residential segregation and promoting trust as well as reciprocity within neighborhoods may help improve the life satisfaction of migrants.

## Introduction

Over the past decades, rural-urban migrants (RUMs) in China have experienced relatively low life satisfaction and limited social integration into urban areas, which have emerged as an important challenge for urban governance and sustainable development [1, 2]. Life satisfaction is a key indicator for assessing the subjective wellbeing of individuals and has been widely applied in existing research [3]. Researchers have extensively examined the determinants of life satisfaction for RUMs, including sociodemographic characteristics (e.g., age and education), economic factors (e.g., labor market conditions), institutional arrangements, and residential environmental factors (e.g., housing type and community social services) [2, 4–6]. As the analytical focus has gradually expanded from the individual level to the spatial context, scholars have paid more attention to examining variations in life satisfaction from the perspectives of community and neighborhood environments [7]. Among various neighborhood factors, residential segregation has been identified as a potentially important contributor to health and wellbeing disparities across social groups.

Residential segregation refers to the physical separation of different groups in residential space based on social categories such as class, race/ethnicity, or religion [8, 9]. Massey and Denton conceptualize residential segregation as a multidimensional phenomenon involving evenness, exposure, concentration, centralization, and clustering, rather than as a single form of uneven spatial distribution [8]. Among these dimensions, exposure is particularly important for understanding migrants’ everyday social life, because it reflects the degree of potential contact or interaction between minority and majority group members in residential space. In the Chinese context, residential segregation mainly occurs between RUMs and local urban residents. A higher proportion of RUMs within a neighborhood implies fewer opportunities for daily contact with local urban residents, which may hinder migrants’ integration into urban society [10]. Therefore, examining residential segregation is important for understanding how neighborhood environments are linked to RUMs’ life satisfaction and social wellbeing.

Existing evidence has mainly come from racially segregated communities in the United States and migrant-concentrated neighborhoods in Europe, where residential segregation is closely associated with unequal resources and lower wellbeing [11, 12]. In China, empirical research on the wellbeing consequences of residential segregation for migrants remains limited. A small number of studies have begun to address this issue. For example, Lu et al. found that residential segregation is associated with physical health among RUMs and that this relationship is partly mediated by social integration and access to public health services [13]. Another study showed that residential segregation may contribute to welfare inequality among older migrants by restricting their access to physical and social resources [14]. These studies suggest that residential segregation is relevant to the physical health and objective welfare of migrants in China. However, they have not directly examined life satisfaction as a subjective evaluative outcome. Therefore, the relationship between residential segregation and life satisfaction among RUMs, as well as the mechanisms underlying this relationship, remains insufficiently understood.

To fill these gaps, this study uses data from a national survey conducted in 2016 to examine the effect of residential segregation on life satisfaction among RUMs. By situating residential segregation in China’s rural-urban institutional context, this study extends research on the neighborhood determinants of migrants’ life satisfaction and clarifies the social mechanisms linking residential segregation to life satisfaction.

### Residential segregation and life satisfaction of RUMs

Theoretically, the effect of residential segregation on migrants’ wellbeing remains debated. From the place stratification perspective, segregated neighborhoods often reflect unequal access to housing, public services, employment opportunities, and neighborhood resources, thereby producing disadvantages for minority or migrant groups [15, 16]. In contrast, the ethnic enclave perspective suggests that migrant or minority concentration may provide emotional support, information exchange, and cultural familiarity, which can buffer disadvantage and improve wellbeing [17, 18].

Unlike residential segregation between people of color and white people in Western societies, which is mainly shaped by racism, residential segregation in China is primarily driven by the combined effects of the urban-rural household registration (*hukou*) system and labor market segmentation it produces [19, 20]. The *hukou* system classifies the population into “urban” and “rural” statuses [21]. Because welfare entitlements and access to government-provided affordable housing are tied to local urban *hukou* status, RUMs often rely on private rental markets [22–24]. Meanwhile, the rural-urban divide institutionalized by the *hukou* system may limit access to education and opportunities for skill formation among RUMs, thereby reinforcing disadvantages in human capital accumulation [25]. These disadvantages concentrate RUMs in low-wage sectors [26, 27], which further constrains housing affordability. Consequently, RUMs tend to gather in informal and low-cost neighborhoods such as urban villages, which are typically characterized by limited public spaces, high population density and inadequate infrastructure [28]. Long-term exposure to these disadvantaged residential environments may intensify social exclusion, discrimination, and psychological stress, thereby undermining life satisfaction [29]. Accordingly, the following hypothesis is proposed:

Hypothesis 1 Residential segregation has a negative impact on life satisfaction among RUMs.

### The role of social capital as a potential mediator

Beyond examining whether residential segregation affects life satisfaction, it is also necessary to clarify the mechanisms through which this relationship emerges. The residential environment can influence life satisfaction by altering daily behaviors and opportunities for social interaction of individuals [30]. Because social capital is generated through sustained daily interactions and social networks [31], residential segregation may partly affect life satisfaction of migrants by shaping the formation and quality of their social capital. Social capital includes structural social capital, reflected in observable networks and participation, and cognitive social capital, reflected in trust, reciprocity, and shared norms [32]. Given these conceptual distinctions, structural and cognitive social capital should be examined separately to assess their distinct mediating roles.

First, residential segregation influences social capital [33]. With regard to structural social capital, residential segregation may limit opportunities for contact between migrants and local residents as well as other social groups, thereby constraining migrants’ access to diverse social ties [34]. Similar effects are also observed among RUMs in China, constraining the expansion and diversification of their social networks [35]. In terms of cognitive social capital, migrant-concentrated neighborhoods usually have high population mobility and low residential stability, which may weaken sustained social interaction and thereby constrain the formation and maintenance of neighborhood trust as well as reciprocity norms [36]. Thus, residential segregation reduces both the structural and cognitive social capital of RUMs.

Second, social capital is an important determinant of life satisfaction [37]. Notably, different types of social capital shape psychological states and subjective wellbeing of individuals through distinct mechanisms [38]. Empirical studies suggest that positive social interactions with other migrants help individuals to buffer the psychological stress caused by discrimination [39–41], thereby improving their life satisfaction. In addition, cognitive social capital promotes individual subjective wellbeing by strengthening a sense of group identity and neighborhood belonging [42]. Evidence from China further supports these associations [43–45].

In summary, residential segregation may affect the life satisfaction of RUMs through its effects on social capital. To date, few studies have directly examined this mediating process. Montero et al. examined the mediating role of social capital in the association between socioeconomic segregation and life satisfaction among international immigrants in Chile [46], but their findings may not be directly generalized to RUMs in China because of differences in the study population and in the socioeconomic contexts of the two countries. This leaves unclear whether social capital also mediates the relationship between residential segregation and life satisfaction among RUMs. Accordingly, the following hypotheses are proposed:

Hypothesis 2a Residential segregation reduces life satisfaction of RUMs by decreasing their structural social capital.

Hypothesis 2b Residential segregation reduces life satisfaction of RUMs by decreasing their cognitive social capital.

**FIGURE 1:**
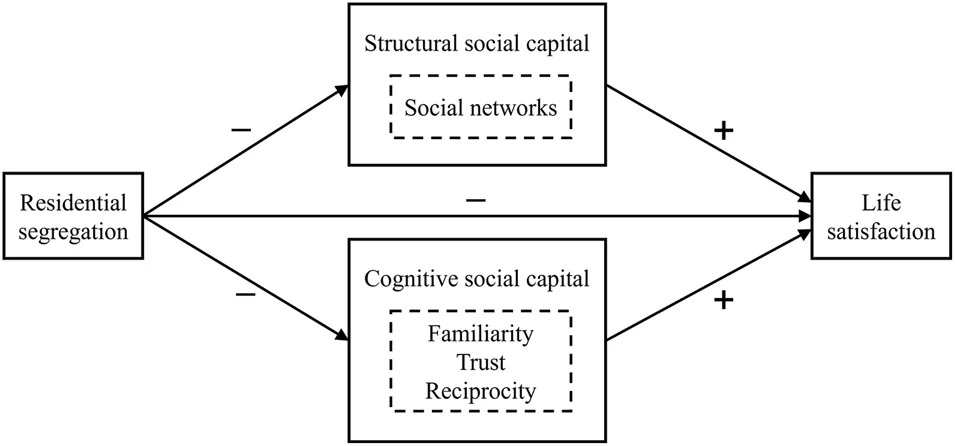
Conceptual framework of the impact of residential segregation on life satisfaction of rural-urban migrants (China, 2026).

Based on the above theoretical arguments, the conceptual framework of the current study is presented in Figure 1.

## Methods

### Data source and participants

This study used data from the 2016 China Labor-force Dynamics Survey (CLDS2016). The CLDS2016, initiated by Sun Yat-sen University, is a large-scale and nationally representative survey of the Chinese labor force, which collected data at the individual, household, and neighborhood levels. The CLDS2016 covered 29 provinces and municipalities in China. All labor force members within sampled households were eligible to participate. The survey adopted a multistage, stratified cluster sampling design with probability proportional to size (PPS). Following the International Labour Organization definition, the CLDS2016 included respondents aged 15–64 years, as well as employed individuals aged 65 years and above. A total of 20,959 valid observations were collected, providing a representative overview of the Chinese labor force. After data cleaning, 1,255 valid cases were retained for analyses. For the purposes of this study, the analytical sample was restricted to RUMs. Respondents were required to have resided in the destination location and hold a non-local rural *hukou*.

### Measures

#### Life satisfaction

Respondents were asked: “Overall, are you satisfied with your life?” Responses were recorded on a five-point Likert scale ranging from “Strongly disagree = 1” to “Strongly agree = 5”, with higher scores indicating higher levels of life satisfaction. The validity of the single-item life satisfaction measure has been tested in previous studies [47]. In addition, this measure has been commonly used in studies of Chinese RUMs [4, 48].

#### Residential segregation

The core independent variable in this study was residential segregation. Following prior research that operationalized residential segregation using the composition of co-ethnic groups or migrants within residential neighborhoods [49, 50], this study measured residential segregation as the proportion of RUMs in the total population of each respondent’s residential neighborhood. This measure was calculated using neighborhood questionnaire data on the total resident population and the number of RUMs in each neighborhood. The resulting ratio ranges from 0 to 1, with higher values indicating a higher level of migrant presence in the neighborhood, which is interpreted in this study as a higher level of residential segregation among RUMs.

#### Social capital

Social capital includes two types: structural social capital and cognitive social capital. Structural social capital was measured based on respondents’ answers regarding the number of social network ties: “how many close friends/acquaintances in the local area can you rely on for support and assistance?” Given the presence of a small number of extreme values and the meaningful value of zero (no local social networks), this variable was winsorized at the 99th percentile and treated as a count variable.

Cognitive social capital was measured using three dimensions: neighborhood familiarity, neighborhood trust, and neighborhood reciprocity. The corresponding survey questions asked respondents about their familiarity with neighbors, their trust in neighbors, and the extent of mutual assistance within the neighborhood. All items were measured on a five-point Likert scale. Factor analysis was conducted to construct a composite index of cognitive social capital. Reliability analysis showed that Cronbach’s α = 0.79 and KMO = 0.71, supporting the suitability of factor analysis. For ease of interpretation, the resulting factor scores were rescaled to a range of 1–5. Ultimately, both composite cognitive social capital index and each dimension were included in the analyses.

#### Covariates

A series of demographic covariates were controlled for in the models: age, gender (Male/Female), educational attainment (No formal schooling/Primary school/Junior high school/Senior high school/College and above), marital status (Unmarried/Married), personal income (natural logarithm), employment status (Unemployed/Employed), self-rated health, and duration of residence. Due to data limitations in CLDS2016, duration of residence was operationalized as a binary variable (Less than 6 months/More than 6 months).

### Analytical strategies

All statistical analyses were conducted using Stata version 17. First, descriptive statistics were provided to summarize life satisfaction, residential segregation, and social capital (both structural and cognitive) as well as the socio-demographic characteristics of respondents. Subsequently, ordinary least squares (OLS) regression was used to assess the impact of residential segregation on life satisfaction. On account of the hierarchical structure of the data (neighborhood and individual) and the relatively small number of neighborhood-level units, the explanatory power of Hierarchical Linear Models was limited. Therefore, OLS regression with neighborhood-level cluster-robust standard errors was applied. This approach accounts for potential intra-neighborhood correlation while maintaining the individual-level analytical framework.

Despite controlling for several demographic characteristics in the baseline model, potential endogeneity issues may remain. To address this, an instrumental variable (IV) was constructed following Lu et al. [13]. Specifically, for each respondent, the IV was defined as the average level of residential segregation among other RUMs who (i) had the same provincial *hukou*, (ii) resided in the same type of neighborhood (e.g., enterprise compounds, old residential areas, apartment compounds, etc.), and (iii) worked in the same type of region (e.g., city, county, town, etc.). The respondent’s own residential segregation level was excluded from this calculation. To retain as much sample information as possible, respondents with missing values on the IV were retained in the OLS models and mediation analyses but were excluded from the IV analysis. Therefore, the IV analysis was based on 1115 observations.

This instrument is expected to satisfy the relevance and the exclusion restriction. Regarding relevance, RUMs from the same province are more likely to rely on relatives and acquaintances when searching for housing, which may lead them to cluster in the same residential areas. Migrants working in the same type of work locations are also more likely to concentrate in the same areas [51]. As a result, RUMs living in the same type of neighborhoods tend to be exposed to comparable residential environments. Therefore, the residential segregation levels of peer RUMs are expected to be correlated with the respondent’s own residential segregation level. Regarding the exclusion restriction, although the residential segregation level of peer groups may be associated with the segregation level experienced by respondents, it is unlikely to directly affect respondents’ own life satisfaction after controlling for a range of covariates related to life satisfaction.

Finally, the mediating effects of social capital were examined. Following the principles of mediation analysis [52], the analysis was conducted in three steps. The empirical models are specified in Equations 1-3:

$${LS}_{i}=\alpha +\beta {RS}_{i}+\gamma Z_{i}+\varepsilon_{i}$$ (1)

$$M_{i}=\alpha_{0}+\beta_{2}{RS}_{i}+\gamma Z_{i}+\varepsilon_{i}$$ (2)

$${LS}_{i}=\alpha +\beta_{3}{RS}_{i}+\delta M_{i}+\gamma Z_{i}+\varepsilon_{i}$$ (3)

where ${LS}_{i}$ denotes the life satisfaction of i th RUM; ${RS}_{i}$ denotes the independent variable of residential segregation; $M_{i}$ represents either structural social capital (e.g., social networks) or cognitive social capital (e.g., familiarity, trust or reciprocity); $Z_{i}$ denotes the control variables for socio-demographic characteristics; $\varepsilon_{i}$ is an error term.

## Results

### Descriptive statistics

Table 1 presents the descriptive statistics of the sample (N = 1,255). The mean life satisfaction score is 3.58 and the average level of residential segregation is 0.34. With regard to social capital, the mean value of structural social capital is 7.05 in a range of 0–60, indicating a generally low level. The average score of cognitive social capital is 3.01, suggesting a moderate level. For specific dimensions of cognitive social capital, the mean scores for neighborhood familiarity, trust and reciprocity are 3.07, 3.21 and 2.70, respectively.

**TABLE 1:** Descriptive statistics of variables (China, 2026).

Variables	Study sample (n = 1,255)			
​	Mean ± SD	Min	Max	Percentage
Life satisfaction	3.58 ± 0.97	1	5	​
Residential segregation	0.34 ± 0.28	0	0.96	​
Structural social capital	7.05 ± 9.47	0	60	​
Cognitive social capital	3.01 ± 0.83	1	5	​
Familiarity	3.07 ± 1.08	1	5	​
Trust	3.21 ± 0.86	1	5	​
Reciprocity	2.70 ± 1.07	1	5	​
Age	37.94 ± 11.44	15	83	​
Income (log)	9.84 ± 2.38	0	13.82	​
Health	3.84 ± 0.86	1	5	​
Gender	​	​	​	​
Male	​	​	​	50.28%
Female	​	​	​	49.72%
Marriage	​	​	​	​
Unmarried	​	​	​	18.33%
Married	​	​	​	81.67%
Education	​	​	​	​
No formal schooling	​	​	​	4.70%
Primary school	​	​	​	20.88%
Junior high school	​	​	​	43.82%
Senior high school	​	​	​	19.28%
College and above	​	​	​	11.31%
Occupation	​	​	​	​
Unemployed	​	​	​	8.84%
Employed	​	​	​	91.16%
Duration of residence	​	​	​	​
Less than 6 months	​	​	​	10.92%
More than 6 months	​	​	​	89.08%

Continuous variables are reported means, standard deviations, and minimum/maximum values. Categorical variables are reported percentages.

### Regression analyses

Model 1 in Table 2 demonstrates the impact of residential segregation on life satisfaction after controlling for relevant demographic variables and accounting for clustering at the neighborhood level. It is indicated that residential segregation (β = −0.246, p < 0.05) exhibits a statistically significant and negative impact on life satisfaction among RUMs. A higher level of residential segregation is related to lower life satisfaction.

**TABLE 2:** The effect of residential segregation on life satisfaction (China, 2026).

​	Model 1: OLS	Model 2: 2SLS-first stage	Model 3:2SLS-second stage
Variables	Life satisfaction	Residential segregation	Life satisfaction
Residential segregation	−0.246*	​	−0.385*
​	(0.123)	​	(0.151)
Instrumental variables	​	0.905***	​
​	​	(0.027)	​
Control variables	Yes	Yes	Yes
Constant	2.303***	−0.023	2.501***
​	(0.263)	(0.073)	(0.349)
K-P LM-stat	​	331.164***	
K-P F-stat	​	1,109.265 [16.38]	
*R* ^2^	0.067	​	
Observations	1,255	1,115	1,115

Instrumental variables are the average level of residential segregation among RUMs; The other control variables include age, gender, education, marriage, income, occupation, self-rated health, and duration of residence; Robust standard errors are shown in parentheses; *p < 0.05, **p < 0.01, ***p < 0.001.

The first-stage regression results in Table 2 (Model 2) show that the IV is significantly correlated with residential segregation. The F-statistic is far above the Stock-Yogo 10% maximal IV size critical value of 16.38, indicating that the hypothesis of a weak IV is rejected. The second-stage regression (Model 3) indicates that residential segregation significantly reduces life satisfaction of RUMs by 0.385 points, or 10.75% of the average level. These findings suggest that the negative regression results remain robust after accounting for potential endogeneity. Hypothesis 1 is supported.

### Mediation analyses

To further understand the mechanisms through which residential segregation affects the life satisfaction of RUMs, this study investigates the mediating role of social capital.

As shown in Table 3, Models 4 and 5 estimate the effects of residential segregation on the two types of social capital, respectively. Model 4 indicates that residential segregation has no statistically significant effect on structural social capital (p > 0.1). In other words, the necessary condition for a mediating effect is not satisfied. Therefore, the mediation effect of structural social capital is not supported. By contrast, we find that residential segregation significantly reduces cognitive social capital (β = −0.336, p < 0.01) in Model 5. Model 7 incorporates cognitive social capital as a mediating variable. Consistent with our expectations, the coefficient of residential segregation is attenuated from −0.246 in the baseline model (Model 1 in Table 2) to −0.194, suggesting that cognitive social capital may serve as a mediator. Only Hypothesis 2b is supported.

**TABLE 3:** Results of the mediation analyses for structural and cognitive social capital (China, 2026).

​	Model 4: NB	Model 5: OLS	Model 6: OLS	Model 7: OLS
Variables	Structural social capital	Cognitive social capital	Life satisfaction	Life satisfaction
Residential segregation	0.232	−0.336**	−0.252*	−0.194
​	(0.227)	(0.113)	(0.123)	(0.124)
Structural social capital	​	​	0.004	​
​	​	​	(0.003)	​
Cognitive social capital	​	​	​	0.155***
​	​	​	​	(0.037)
Control variables	Yes	Yes	Yes	Yes
Constant	1.381**	2.149***	2.295***	1.969***
​	(0.494)	(0.276)	(0.264)	(0.274)
*R* ^2^	0.009	0.078	0.069	0.084
Observations	1,255	1,255	1,255	1,255

NB is Negative Binomial regression; Pseudo R^2^ is shown in Model 4; Robust standard errors are shown in parentheses; *p < 0.05, **p < 0.01, ***p < 0.001.

Next, we further evaluated the role of different dimensions of cognitive social capital. As shown in Table 4, residential segregation has a significant negative effect on trust and reciprocity, while its effect on familiarity (p > 0.05) is not statistically significant (Models 8-10). Since familiarity does not satisfy the necessary condition for a mediation effect either, its mediating effect is not supported. Models 12 and 13 include trust and reciprocity as mediators, respectively. The results show that the direct effects of residential segregation on life satisfaction (Trust β = −0.215; Reciprocity β = −0.205) are reduced compared with the baseline model. These findings suggest that only trust and reciprocity mediate the relationship between residential segregation and life satisfaction within cognitive social capital.

**TABLE 4:** Results of the mediation analyses for familiarity, trust, and reciprocity (China, 2026).

​	Model 8: OLS	Model 9: OLS	Model 10: OLS	Model 11: OLS	Model 12: OLS	Model 13: OLS
Variables	Familiarity	Trust	Reciprocity	Life satisfaction	Life satisfaction	Life satisfaction
Residential segregation	−0.272	−0.267**	−0.487**	−0.213	−0.215	−0.205
​	(0.141)	(0.091)	(0.164)	(0.123)	(0.124)	(0.124)
Familiarity	​	​	​	0.120***	​	​
​	​	​	​	(0.028)	​	​
Trust	​	​	​	​	0.115**	​
​	​	​	​	​	(0.033)	​
Reciprocity	​	​	​	​	​	0.085**
​	​	​	​	​	​	(0.027)
Control variables	Yes	Yes	Yes	Yes	Yes	Yes
Constant	1.852***	2.483***	2.027***	2.080***	2.016***	2.130***
​	(0.341)	(0.276)	(0.352)	(0.273)	(0.271)	(0.265)
*R* ^2^	0.077	0.053	0.055	0.084	0.077	0.076
Observations	1,255	1,255	1,255	1,255	1,255	1,255

Robust standard errors are shown in parentheses; *p < 0.05, **p < 0.01, ***p < 0.001.

## Discussion

This study aims to examine the impact of residential segregation on the life satisfaction of RUMs in China and to explore the mediating roles of structural and cognitive social capital. The results demonstrate that residential segregation has a significant and robust negative effect on life satisfaction among RUMs. This finding aligns with previous studies conducted in Western contexts [53]. Residential segregation represents a key mechanism through which group-based inequalities are reproduced, giving rise to a wide range of social outcomes including disparities in life satisfaction and health [11, 54].

Different types of social capital exhibit heterogeneous mediating effects in the relationship between residential segregation and life satisfaction. The results indicate that only cognitive social capital acts as a significant mediator, whereas structural social capital does not demonstrate the expected mediating effect. For structural social capital, residential segregation is not significantly associated with social network ties among RUMs (Model 4 in Table 3). This result is inconsistent with our expectation. A possible explanation is that RUMs tend to establish relatively dense internal networks based on geographic proximity or occupational ties in highly segregated communities, such as fellow relationships or co-worker groups [55]. However, these strong ties are highly homogeneous and are difficult to transform into bridging social capital across groups [56]. As a result, they provide limited access to diverse information, institutional resources, or upward mobility opportunities, thus contributing little to overall life satisfaction. Moreover, homogeneous social networks may generate pressures related to reciprocal obligations and social comparison, which can increase psychological stress and pose potential risks to mental health [57, 58].

By contrast, cognitive social capital plays a more effective role in mitigating the negative impact of residential segregation on life satisfaction. Neighborhood-level cognitive social capital influences subjective evaluations by providing emotional support, strengthening community cohesion and alleviating feelings of social isolation [59]. A study focusing on older migrants in China shows that the neighborhood constitutes the core space of daily life after migration [43]. Migrants often cope with life stressors and adverse events by establishing neighborhood ties within their residential locations. In the process of social interaction, neighborhood trust and reciprocity help reshape expectations and self-perceptions of RUMs, thereby affecting their overall assessment of life conditions [60]. Therefore, compared with social networks, cognitive social capital is more capable of enhancing the life satisfaction of RUMs through socio-psychological mechanisms.

Further analyses reveal obvious differences among the internal dimensions of cognitive social capital in mediating the effects of residential segregation. The findings indicate that neighborhood familiarity does not exert a significant mediating effect between residential segregation and life satisfaction, whereas trust and reciprocity constitute the key mediating pathways. One possible explanation is that neighborhood familiarity mainly reflects the frequency of contact or superficial interaction with neighbors, and does not necessarily imply stable mechanisms of resource exchange or emotional investment. As a result, such ties are less likely to be converted into social resources that alleviate psychological stress or provide substantive support [61, 62]. Their capacity to buffer the negative effects of residential segregation is therefore limited.

By contrast, trust and reciprocity are usually embedded in higher quality social relationships, in which individuals develop relatively stable patterns of resource exchange and emotional commitment through sustained interactions. These characteristics enable such relationships to provide more effective and substantive support under conditions of uncertainty or stress [63]. Therefore, maintaining neighborhood trust and reciprocal practices remains crucial for life satisfaction of RUMs living in residentially segregated environments.

Although these findings highlight the explanatory role of cognitive social capital, the observed relationship between residential segregation and life satisfaction may also be influenced by other mechanisms or potential confounders. Residential environments may differ in commuting burden, sense of place, residential satisfaction, and perceived social exclusion [64–66], all of which can influence migrants’ subjective evaluations of their life conditions. Therefore, cognitive social capital should be understood as one important pathway linking residential segregation to life satisfaction, rather than as the only possible explanation.

Taken together, this study has several strengths. First, this study focuses on the impact of residential segregation on life satisfaction among RUMs in China, thereby extending prior research that has mainly examined objective welfare outcomes such as physical health and access to resources. Second, the findings supplement and refine the simplified assumption that social capital is inherently beneficial. They suggest that the qualitative attributes of social capital (especially trust and reciprocity) are more explanatory than frequency of contact or the scale of social networks in explaining the relationship between residential segregation and life satisfaction for migrant populations. Third, this study situates residential segregation among RUMs within China’s rural-urban institutional context. Rather than examining residential segregation only through the lens of racial or ethnic separation, it focuses on a form of residential segregation linked to the *hukou* system and the labor market segmentation it produces, thereby clarifying how residential segregation may contribute to the production and reproduction of wellbeing inequalities. These findings offer theoretical implications for understanding neighborhood-level social integration and practical implications for designing targeted public policies for migrants.

With the acceleration of urbanization and increasing population mobility in China, reducing residential segregation among RUMs and improving their wellbeing has become an urgent policy challenge. Urban planning and housing policies should aim to mitigate residential segregation and promote the construction of socially mixed neighborhoods. Meanwhile, a fairer and more livable environment for RUMs can be achieved through the provision of affordable housing and the renovation of urban villages. However, policies should focus not only on physical spatial integration but also on fostering high-quality social relationships centered on trust and reciprocity among RUMs. Accordingly, urban community governance should shift from an emphasis on the quantity of interactions to a focus on interaction quality. Through institutionalized and sustained governance arrangements, such efforts can reduce social distance and group boundaries as well as provide long-term institutional support for enhancing the life satisfaction of migrants.

### Limitations

This study has several limitations. First, although IV was employed to address potential endogeneity, the cross-sectional data inherently constrains the ability to establish causal relationships rigorously. Second, due to data limitations, the measurement of social networks remains relatively simple and fails to capture additional network characteristics, which may lead to an underestimation of network effects or difficulties in identifying them. Third, although single-item measurement of life satisfaction has been shown to have reliability and validity in general adult samples, evidence on its validity among Chinese populations or RUMs remains limited. Future research should incorporate panel data, more detailed measurements of social networks and larger sample sizes to further validate the proposed mechanisms and enhance the robustness of the findings.

### Conclusion

Based on CLDS2016 data, this study examines the impact of residential segregation on the life satisfaction of rural-urban migrants, focusing on the mediating role of social capital. The findings demonstrate a significant negative association between residential segregation and life satisfaction of migrants. This relationship is mediated by cognitive social capital, especially neighborhood trust and reciprocity. These results underscore the importance of addressing residential segregation while simultaneously fostering neighborhood social capital. Such efforts are essential for enhancing the life satisfaction of migrants, facilitating their social integration and supporting sustainable urbanization in China.
